# The DNA damage response is developmentally regulated in the African trypanosome

**DOI:** 10.1016/j.dnarep.2018.11.005

**Published:** 2019-01

**Authors:** J.P. Vieira-da-Rocha, D.G. Passos-Silva, I.C. Mendes, E.A. Rocha, D.A. Gomes, C.R. Machado, R. McCulloch

**Affiliations:** a Departamento de Bioquímica e Imunologia, ICB, Universidade Federal de Minas Gerais, Av. Antônio Carlos, 6627, Caixa Postal 486, Belo Horizonte, 30161-970, MG, Brazil; b The Wellcome Centre for Molecular Parasitology, College of Medical, Veterinary and Life Sciences, Institute of Infection, Immunity and Inflammation, University of Glasgow, Sir Graeme Davies Building, 120 University Place, Glasgow, G12 8TA, UK

**Keywords:** BER, base excision repair, BSF, bloodstream form, DR, direct repair, DSBs, double strand breaks, HR, homologous recombination, ICL, interstrand crosslink, kDNA, kinetoplast DNA, MMR, mismatch repair, MMS, methyl methanesulfonate, nDNA, nuclear DNA, NER, nucleotide excision repair, NHEJ, non-homologous end joining, PCF, procyclic form, PI, propidium iodide, TLS, translesion synthesis, VSG, variant surface glycoprotein, Trypanosome, Life cycle, DNA damage response, Replication, Repair, Cell cycle

## Abstract

Genomes are affected by a wide range of damage, which has resulted in the evolution of a number of widely conserved DNA repair pathways. Most of these repair reactions have been described in the African trypanosome *Trypanosoma brucei*, which is a genetically tractable eukaryotic microbe and important human and animal parasite, but little work has considered how the DNA damage response operates throughout the *T. brucei* life cycle. Using quantitative PCR we have assessed damage induction and repair in both the nuclear and mitochondrial genomes of the parasite. We show differing kinetics of repair for three forms of DNA damage, and dramatic differences in repair between replicative life cycle forms found in the testse fly midgut and the mammal. We find that mammal-infective *T. brucei* cells repair oxidative and crosslink-induced DNA damage more efficiently than tsetse-infective cells and, moreover, very distinct patterns of induction and repair of DNA alkylating damage in the two life cycle forms. We also reveal robust repair of DNA lesions in the highly unusual *T. brucei* mitochondrial genome (the kinetoplast). By examining mutants we show that nuclear alkylation damage is repaired by the concerted action of two repair pathways, and that Rad51 acts in kinetoplast repair. Finally, we correlate repair with cell cycle arrest and cell growth, revealing that induced DNA damage has strikingly differing effects on the two life cycle stages, with distinct timing of alkylation-induced cell cycle arrest and higher levels of damage induced death in mammal-infective cells. Our data reveal that *T. brucei* regulates the DNA damage response during its life cycle, a capacity that may be shared by many microbial pathogens that exist in variant environments during growth and transmission.

## Highlights

• DNA repair kinetics evaluated in *T. brucei* nuclear and mitochondrial genomes.
• Higher efficiency of DNA repair in *T. brucei* cells from the mammal than the tsetse.
• Differing cell cycle and survival responses to DNA damage in two *T. brucei* cell types.
• Mitochondrial DNA repair is active in *T. brucei* and can involve RAD51.

## 1 Introduction

**Fig. 1:**
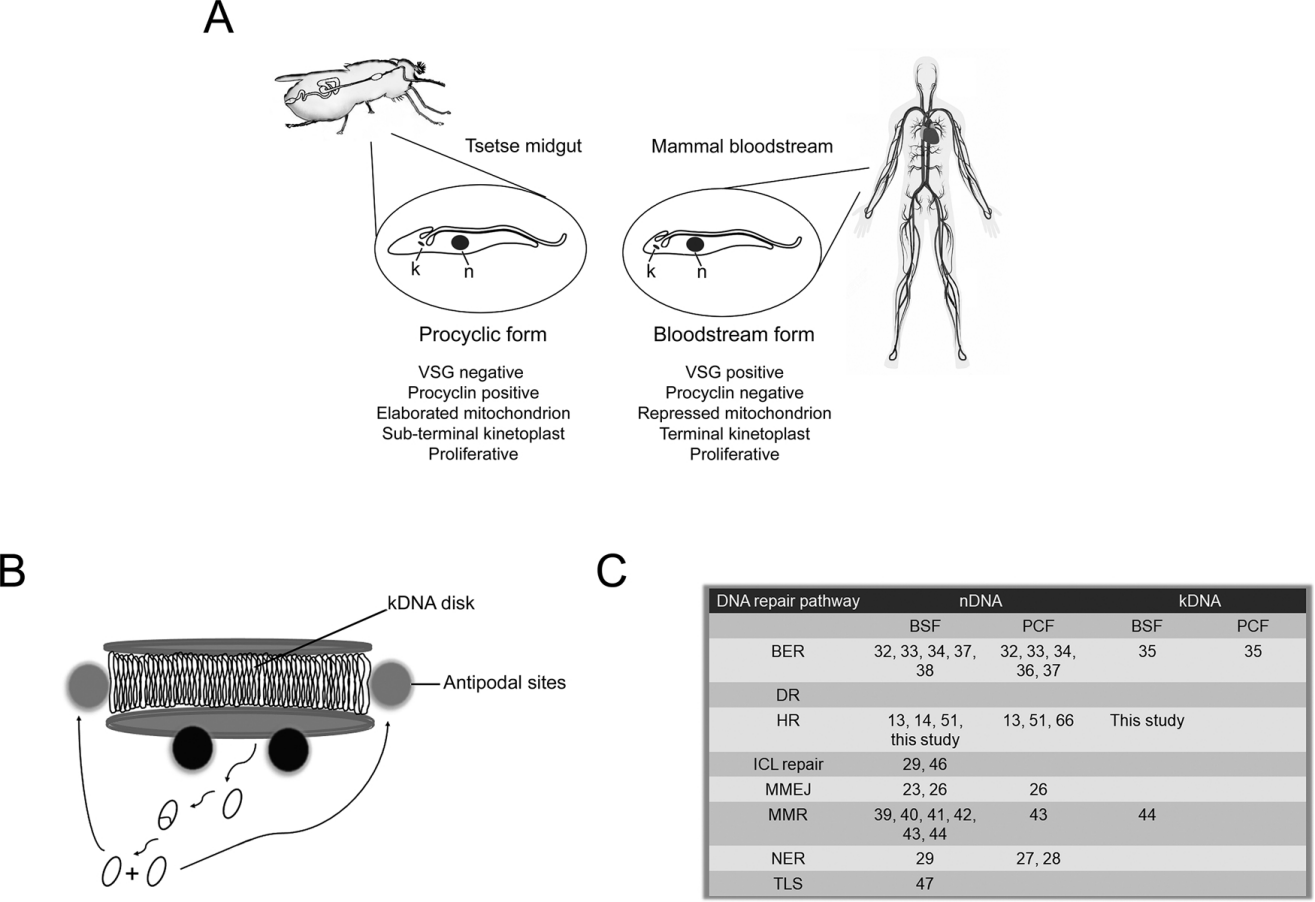
***Trypanosoma brucei* life cycle stages examined in this study, and aspects of their genome maintenance A)** The two life cycle forms used in this study are shown: the replicative long slender bloodstream form (BSF) and the replicative procyclic form (PCF), which are found, respectively, in the mammalian bloodstream and tsetse fly midgut. Cellular hallmarks of the two life cycle stage are presented below their respective cartoons (k, kinetoplast; n, nucleus; VSG, variant surface glycoprotein). **B)** Current model of kinetoplast structure and replication in *T. brucei* [83]. Minicircles and maxicircles are concatenated and organized parallel to the axis of the kinetoplast disk. Covalently closed minicircles (circular shapes) are detached from the kinetoplast disk to initiate replication as θ structures (θ shapes). DNA polymerases, as well as other proteins involved in kDNA replication, are represented by black spheres. After replication, gapped or nicked progeny minicircles migrate to antipodal sites (grey spheres), where gap filling by DNA polymerase β, sealing by ligase kβ, and linkage to the kDNA network by topoisomerase II occurs. Further gap filling and sealing can occur at the kinetoplast disk by the action of DNA polymerase β-PAK and ligase kα. **C)** Overview of DNA repair pathways identified and/or characterized in the nucleus (nDNA) or kinetoplast (kDNA) of BSF or PCF trypanosomes; DNA repair pathways are abbreviated as in the text, and numbers are references cited in the text. Cartoons were modified from [[83], [84], [85]].

*Trypanosoma brucei* is the causative agent of sleeping sickness in humans and nagana in livestock. The parasite has a complex life cycle, undergoing multiple changes as it develops within and transmits between mammal hosts and the testse fly vector. Such changes include alterations in metabolism [1], composition of surface proteins [2], and organelle organization inside the cell body [3]. Within testse flies (*Glossina* genus), *T. brucei* differentiates between replicative and non-replicative forms in both the digestive system and in the salivary glands [4]. Currently, only replicative procyclic forms (PCF) cells from the fly midgut are routinely grown and genetically manipulated in culture (Fig. 1A). Non-replicative metacyclic form cells in the tsetse salivary gland establish infections in mammals, after fly feeding, by differentiating into the replicative long slender bloodstream form (BSF), which can also be routinely cultured and modified (Fig. 1A). BSF cell survival in the mammal critically depends on expression of a ‘coat’ composed of a single variant surface glycoprotein (VSG), which is periodically switched to an antigenically distinct VSG type to thwart clearance by the host adaptive immune response [[5], [6], [7]]. In contrast, PCF cells do not require VSG antigenic variation and, instead, they express different forms of procyclin on their surface [8]. Despite these differences in the cell surface proteome, allied to alterations in cell biology and metabolism, both PCF and BSF cells appear to function to establish and maintain infections through growth by mitotic division. Nonetheless, comparisons of the two life cycle stages suggest differences in cell cycle timing and in checkpoints [9,10]. What is less clear is if these growth differences extend to changes in the use or execution of the DNA damage response, which is critical for the successful transmission of intact, functional genomes from parent to progeny. In all kinetoplastids, maintenance of the unusual mitochondrial genome, termed the kinetoplast (Fig. 1B, discussed below), is likely also to require DNA repair pathways, which are poorly characterized relative to the nucleus (Fig. 1C).

Antigenic variation has provided a motivation for understanding the DNA damage response in *T. brucei*, since the available evidence suggests this process is intimately linked to homologous recombination (HR) [11], which is universally conserved throughout life and thought to mainly act in the repair of DNA double strand breaks (DSBs) and replication fork stalls [12]. A switch in the VSG coat is primarily executed by recombination reactions that replace the VSG gene present in actively transcribed VSG expression site with a VSG gene from a silent archive of >1000 genes or pseudogenes [6]. Most VSG recombination occurs by gene conversion, and mutation of many components of the HR pathway, including RAD51 [13], BRCA2 [14], RAD51-related proteins [15], Topo III-α [16], and a RECQ helicase and putative interactors [17], has been described as altering the rate and profile of VSG gene conversion in BSF *T. brucei*, as well as altering general genome repair. However, these studies have mainly been limited to intact silent VSGs, and little is known about the reaction(s) that can generate novel mosaic VSGs through segmental gene conversion of intact and pseudo VSG genes variation [18]. In addition, most living organisms perform DSB repair not simply by HR, but also by end-joining of the DSB. Most commonly, such direct repair of the broken molecule occurs by non-homologous end joining (NHEJ), which relies on a dedicated machinery, including the DSB binding factor Ku [19]. DSB repair in *T. brucei* and related kinetoplastid parasites appears to rely mainly on HR, perhaps because the parasites lack NHEJ: key factors are missing [[20], [21], [22]], and targeted assays to follow repair of an induced DSB (by I-SceI [23] or CRISPR-Cas9 [24,25] cleavage) have failed to detect NHEJ. Microhomology-mediated end-joining (MMEJ; sometimes called alternative NHEJ) can be detected in some cases [23,26], but the conditions in which this reaction is utilized, and the enzymatic machinery involved, have not been extensively investigated. Whether MMEJ contributes to VSG switching is unknown.

Though most experimental efforts have addressed the role of recombination repair in *T. brucei*, further DNA repair pathways operate, or can be predicted to do so (Fig. 1C). Genetic and biochemical studies clearly demonstrate the action of Nucleotide Excision Repair (NER) [[27], [28], [29]], Base Excision Repair (BER) [[30], [31], [32], [33], [34], [35], [36], [37], [38]] and Mismatch Repair (MMR) in *T. brucei* [[39], [40], [41], [42], [43], [44]]. In contrast, further *T. brucei* DNA repair pathways are less well explored, including Direct Reversion Repair (DR) [45], Interstrand Crosslink Repair (ICL repair) [29,46] and Translesion Synthesis (TLS) [47]. In all cases, the potential that trypanosomatid repair pathways might differ from counterpart pathways in model eukaryotes, reflecting parasite-specific biology, is relatively poorly understood. One validated example of such a difference is specialization of NER due to near universal nuclear multigenic transcription, but even here the details remain incompletely resolved [29]. A further potential source of novelty lies in the highly unusual structure of the genome of the single copy trypanosomatid mitochondrion, or kinetoplast (Fig. 1B). The kinetoplast is composed of a network of circular, concatemerized DNA molecules of ∼1 and 23 kb known, respectively, as minicircles and maxicircles. Maxicircles are present in 30–50 copies and have homology with the mitochondrial genome of other eukaryotes, whereas minicircles are present in several thousands of copies and encode RNAs specialized for mitochondrial RNA editing [48]. Mitochondrial targeting of DNA polymerases normally involved in nuclear BER or TLS [48] suggest the action of some repair pathways in the kinetoplastid, or co-option of repair components for replication, but a full description of kinetoplastid repair capacity is lacking.

A further potential source of *T. brucei* and trypanosomatid DNA repair specialization, for which there is limited information, concerns the signaling or regulation of the pathways, and whether the repair mechanisms are differentially modulated throughout the parasite’s developmental programmes. While high throughput phenotyping of *T. brucei* has revealed a range of activities involved in alkylation damage repair [49], such work has so far been limited to BSF cells and only a small number of focused experiments have compared repair in BSF and PCF cells. For instance, TLS DNA Polymerase Kappa (Polκ) and the homolog of Structural Maintenance of Chromosome 1 (SMC1, which promotes chromosomal stability in yeast by channeling HR between sister chromatids) are essential for BSF cells but not for PCF. Conversely, the *T. brucei* homolog of DNA Damage Inducible Protein 1 (DDI1-like) is essential for PCF cells but not for BSF [50]. Deletion of BRCA2, a protein which acts in HR by promoting Rad51 loading onto RPA-coated single-stranded DNA stretches, is non-lethal in both life cycle stages, but has a higher impact on BSF fitness than it has in PCF, since it causes observable chromosomal instability in the former but not in the latter [51]. Moreover, deletion of MSH2, which plays a central role in MMR, generates distinct phenotypes in BSF and PCF cells: where BSF MSH2 null mutants display increased sensitivity to hydrogen peroxide, the same mutants increase PCF cell tolerance to oxidative stress, even though both life stages display MMR deficiency [43].

Though the above data indicate developmental stage variation in *T. brucei* genome stability pathways, it is less clearly understood how these modulations relate to repair kinetics, cell cycle progression or which genome is predominantly affected. Here we have investigated each of these questions. Using highly sensitive, quantitative PCR we measured DNA damage levels in both the nuclear DNA (nDNA) and kinetoplast DNA (kDNA), and in BSF and PCF cells, after exposure to a range of treatments. In doing so, we evaluated, in both life cycle forms and in both genomes, repair kinetics of the different lesions. We also correlated repair kinetics with profiles of cell cycle arrest and survival after DNA damage. We demonstrate that DNA repair occurs in the kinetoplast, with pronounced similarities in repair kinetics relative to the nucleus. In addition, we show that repair kinetics and the cellular response to damage differ dramatically between the different forms of damage examined, and differ dramatically between BSF and PCF cells. Taken together, these results suggest trypanosomatids can differentially regulate the DNA damage response during growth and development.

## 2 Methods

### 2.1 Cell culture and parasite strains

Procyclic forms of *T. brucei* strain Lister 427 were cultivated at 27 °C in SDM-79 media [52], supplemented with 10% heat inactivated fetal bovine serum (Sigma), 0.2% (v/v) of hemin (Sigma) solution at 2 mg mL^−1^ in 0.2 M NaOH, and 1% (v/v) penicillin and streptomycin (Sigma). Bloodstream forms of *T. brucei* strain Lister 427 were maintained at 37 °C in a humidified incubator with 5% CO_2_ in HMI-9 media [53], supplemented with 10% heat inactivated fetal bovine serum (Sigma) and 1% (v/v) penicillin and streptomycin (Sigma). CSB RNAi knockdown and Rad51 knockout BSF strains were generated and described previously in [29] and [51].

### 2.2 Parasite growth curves and treatment with genotoxic agents

BSF and PCF cells at the mid-log phase growth were harvested by 15 min of centrifugation at 3000 *g* at 4 °C and resuspended in the same volume of 1x PBS. For DNA photo-lesion formation, parasites resuspended in 1x PBS were centrifuged again for 15 min at 3000 *g* and 4 °C and the volume of parasite suspension in 1x PBS was adjusted to 5 mL. Next, parasites were spread in a small Petri plate, which was irradiated with 30, 60 and 120 J/m^2^ of UVC light (254 nm) with a Stratalinker® UV Crosslinker (Stratagene). Bulky DNA adducts were induced through exposure to 50, 100, 200, and 400 μM of cisplatin in 1x PBS for one hour. Oxidative DNA damage was induced through treatment with 50 μM of hydrogen peroxide (H_2_O_2_) in 1x PBS for 30 min, and 50 and 100 μM H_2_O_2_ in 1 x PBS for one hour. Alkylating DNA damage was induced by one hour of exposure to 0.5 and 1.5 mM Methyl Methanesulphonate (MMS) in 1x PBS. After genotoxic treatments cells were centrifuged for 15 min at 3000 *g* at 4 °C and then resuspended in conditioned media (i.e the media used in the original culture). Finally, cells were diluted 10 times and parasite growth was monitored daily by hemocytometer counting until parasites reach stationary phase.

### 2.3 Detection of DNA lesions through quantitative PCR

DNA damage was quantified according to previously described approaches in mammalian cells [54] and *T. brucei* [29]. DNA damage was indirectly quantified through quantitative PCR, since the amplification yield is inversely proportional to the amount of DNA lesions. Briefly, large fragments spanning 10 kb of either the *T. brucei* mitochondrial or nuclear genome were PCR amplified, with or without prior growth in the presence of DNA damage induction. The PCR-amplified fragment of mitochondrial DNA was derived from the maxicircle. A small internal fragment comprising 204 bp was used to normalize the amplification of the larger fragments. For induction of damage, the following treatments were used: 100 μM exposure for one hour for cisplatin or hydrogen peroxide treatment; 30 J/m^2^ for UVC exposure; and 1.5 mM for one hour for MMS damage induction. For UVC exposure, parasites were suspended in 5 mL of 1x PBS and spread in a small Petri plate. After DNA damage induction, approximately 1 × 10^8^ cells were collected at 0, 1, 2, 4 and 8 h after each treatment, centrifuged at 3000 *g* and 4 °C for 15 min, and the resulting cell pellet immediately frozen at −80 °C. DNA was extracted from parasite cells with the Blood & Cell Culture DNA Mini Kit (QIAGEN), according to the manufacturer’s instructions for DNA extraction from tissues. DNA was then quantified using PicoGreen dye (Molecular Probes), as described by Santos et al. [54]. PCR was performed from 15 ng of template DNA using the GeneAmp XL PCR Kit (Applied Biosystems) and the resulting amounts of PCR product quantified, again using PicoGreen and as described by Santos et al. [54]. All PCR reactions were carried out only until the logarithmic phase, when the amplification yields are directly proportional to the starting amount of template. The primers used in the amplification reaction of the large fragment in nDNA were qPCR Forward (5′-GTTGCTCACTTTCACCACGTATTCGGGAACCTGT-3′) and qPCRReverse (5′-CCACTGAATGCTGTATCCGGCATTTAGTCGTGTCTATGGG-3’). To PCR-amplify the small nuclear fragment, used as the internal control, the primers qPCRFI (5′-TTACAGCACCCAGGTTTATACCGCACGAAAGTGG-3′) and qPCRReverse were used. To amplify the large fragment from kDNA the primers MtF (5’-TAAGTACAAGAGGAGACAGACGACAGTGTCCACAGCAC-’) and MtR2 (5’-TCGAACGGCTCTTTCTCTCCAGT-3’) were used. The primers MtFI2 (5’-CCAACACTCCATTCCTGTTCACACCGTGATTCTTCTC-3’) and MtR2 were used to amplify the mitochondrial small internal fragment. All primers were purchased from Eurofins MWG Operon.

### 2.4 Cell cycle analysis

Cell cycle analysis was performed by flow cytometry. After DNA damage induction through exposure to 100 μM of cisplatin for one hour or by treatment with 0.5 and 1.5 mM of MMS for one hour, parasites were centrifuged, resuspended in conditioned media and cultivated for recovery at different time points as indicated in results section. At each time point, approximately 0.5–1 × 10^6^ cells were harvested, washed once in 1x PBS and suspended in fixation solution containing 70% methanol and 30% PBS. Cells were then kept at 4 °C overnight to several days, according to each experiment time course. Cells were washed in 1 mL of cold 1x PBS and resuspended in 1 ml of 1 x PBS containing 10 μg ml^−1^ propidium iodide (PI) and 10 μg ml^−1^ RNaseA. Cells were then incubated at 37 °C for 45 min and flow cytometry was performed with a Becton Dickinson FACScan, using detector FL2-A and a total of 10,000 events. Data were analyzed with FlowJo v10^™^ software.

### 2.5 FITC annexin V assay

To perform the FITC Annexin V assay we treated the parasites with cisplatin and MMS. For MMS treatment, parasites were exposed to 0.5, 1.0 and 1.5 mM of MMS for one hour and immediately analyzed by FACS. For cisplatin treatment, cells were incubated for one hour with 100, 200 and 400 μM of cisplatin and analyzed by FACS 24 h after DNA damage induction. For FACS analysis, approximately 0.5–1 × 10^7^ cells were harvested and washed in cold 1x PBS and resuspended in 500 μL of 1x annexin-binding buffer (10 mM HEPES, 140 mM NaCl, 2.5 mM CaCl_2_, pH 7.4), containing 200 ng mL^−1^ of PI and 5 μL of FITC annexin V (Invitrogen). After incubation for 5 min, cells were analyzed by flow cytometry in a total of 5000 events and using detectors for FL1-H and FL2-H, which detect respectively FITC annexin V and PI fluorescence. Control cells used to set up compensation and quadrants were washed in 1x PBS, fixed in 70% ethanol and labeled with IP and/or FITC Annexin V according described above. Data were analyzed using FlowJo v10^™^ software.

### 2.6 Statistical analysis

All statistical analyses were performed with the software GraphPad Prism version 6.01.

## 3 Results

### 3.1 BSF *T. brucei* cells display higher efficiency than PCF cells in repair of DNA adducts induced by cisplatin

**Fig. 2:**
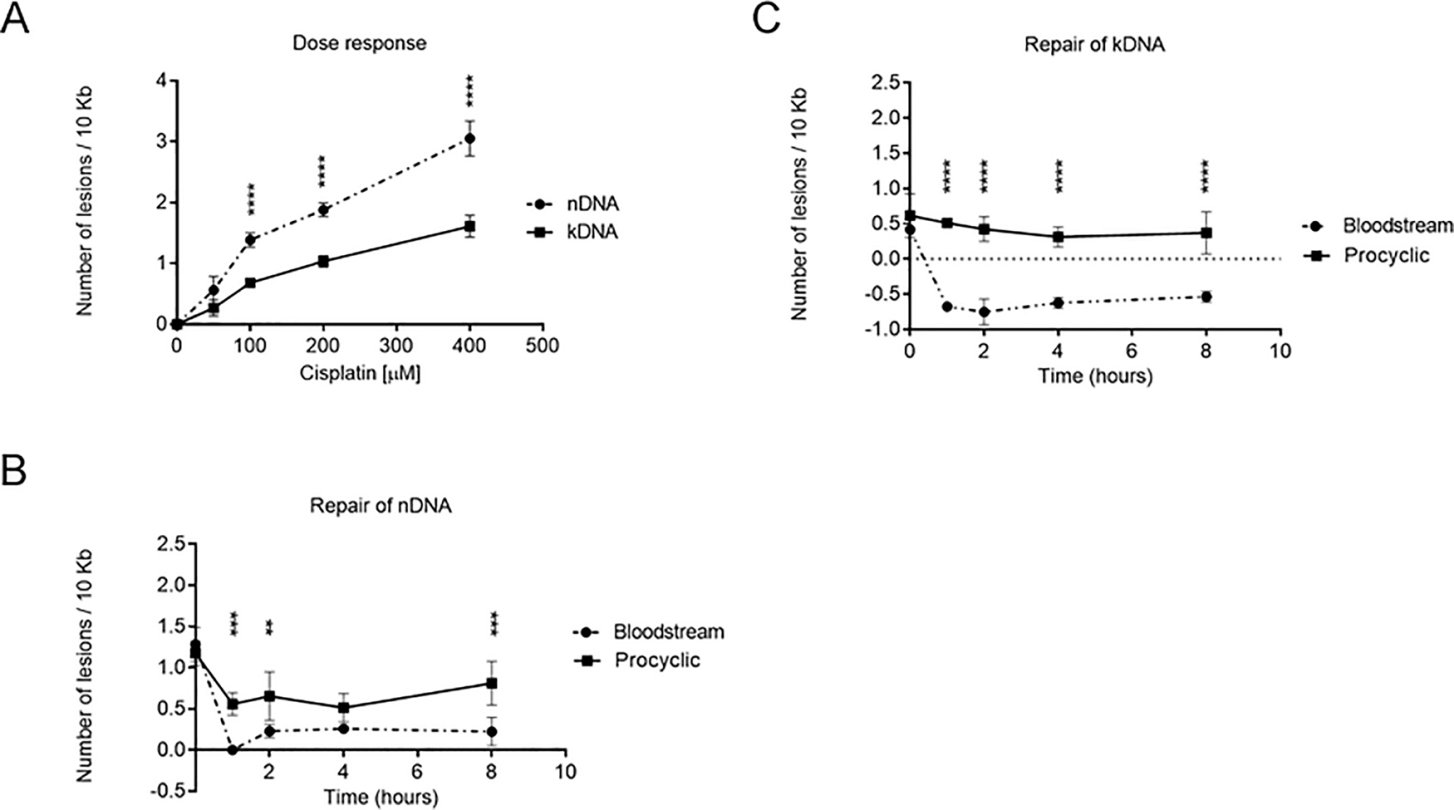
**Induction and repair of cisplatin-induced DNA damage in nuclear DNA (nDNA) and kinetoplast DNA (kDNA). A)** Dose response curve of lesions in nDNA (traced line with circles) and kDNA (full line with squares) of BSF cells as a function of cisplatin doses. Cells were treated with 50 to 400 μM of cisplatin for 1 h. **B)** DNA repair kinetics of nDNA from BSF (traced line with circles) and PCF (full line with squares) cells after treatment with 100 μM of cisplatin for 1 h. **C)** DNA repair kinetics of kDNA from BSF and PCF cells treated as described in (B). Negative values of lesions indicate that the frequency of lesions/10 kb is lower than the basal level. The data were obtained from two independent PCR amplifications derived from each of the two biological duplicates. Data were analyzed using Two-way ANOVA repeated measures with fixed effects for cell type, time, and their interaction. This analysis was followed by Sidak’s multiple comparisons post test. Error bars denote standard deviation and **, ***, and **** mean respectively p values less than 0.01, 0.001, and 0.0001.

We previously showed that BSF *T. brucei* present high efficiency repair of nDNA damage induced by cisplatin, and that the proficiency of removal of cisplatin-induced adducts in BSF cells relies on transcription-coupled NER [29]. Here, we used the same approach to measure the level of damage induction and kinetics of repair in PCF nDNA after exposure to cisplatin. In addition, we monitored damage induction and repair in the kDNA of both *T. brucei* life stages. We first performed a dose response curve in BSF cells, with doses ranging from 50 to 400 μM of cisplatin for one hour, to select which dose will induce >1 lesion/10 kbp (Fig. 2A). Treatment with 100 μM of cisplatin induced ∼1.5 lesions/10 kbp after 1 h in the nDNA of both life cycle forms, without inducing high levels of cell death 10 h after treatment (Figs. 2A and S1 A). However, the levels of adducts induced by cisplatin were higher in nDNA than in kDNA at all concentrations tested, with ∼0.5 lesions/10 kbp of kDNA at 100 μM (Fig. 2A). We next compared the kinetics of cisplatin lesion repair in both life stages of *T. brucei*. Within the first hour post-treatment BSF cells removed essentially all lesions in the regions analyzed in both the nDNA (Fig. 2B) and kDNA (indeed, at this time point kDNA lesion levels were even lower than the basal level of kDNA damage; Fig. 2C). Conversely, in PCF cells only half of nDNA lesions were removed throughout the entire 8 h of time course (Fig. 2B), and the level of lesions in the kDNA did not reduce to basal levels of damage (Fig. 2C). Therefore, though both life stages of *T. brucei* can repair cisplatin-induced adducts, lesion removal occurs from either the nDNA or kDNA more efficiently in BSF cells than in PCF. Previously, we demonstrated that the rapid repair of cisplatin in BSF *T. brucei* contrasts with the slow kinetics of UVC-induced DNA damage repair [29]. To ask if slow UVC lesion repair is only seen in BSF cells, we compared nDNA and kDNA repair kinetics in the two life cycle stages (Fig. S2). In both BSF and PCF cells UVC lesion removal was either slower than that of similar levels of cisplatin lesions (nDNA), or was not detectable in the time course (kDNA). Thus, both life cycle forms of the parasite have a limited capacity to repair these levels of nuclear UVC damage and an even lower capacity to repair UVC damage to the kinetoplast.

### 3.2 Repair of oxidative damage is more effective in BSF than in PCF

**Fig. 3:**
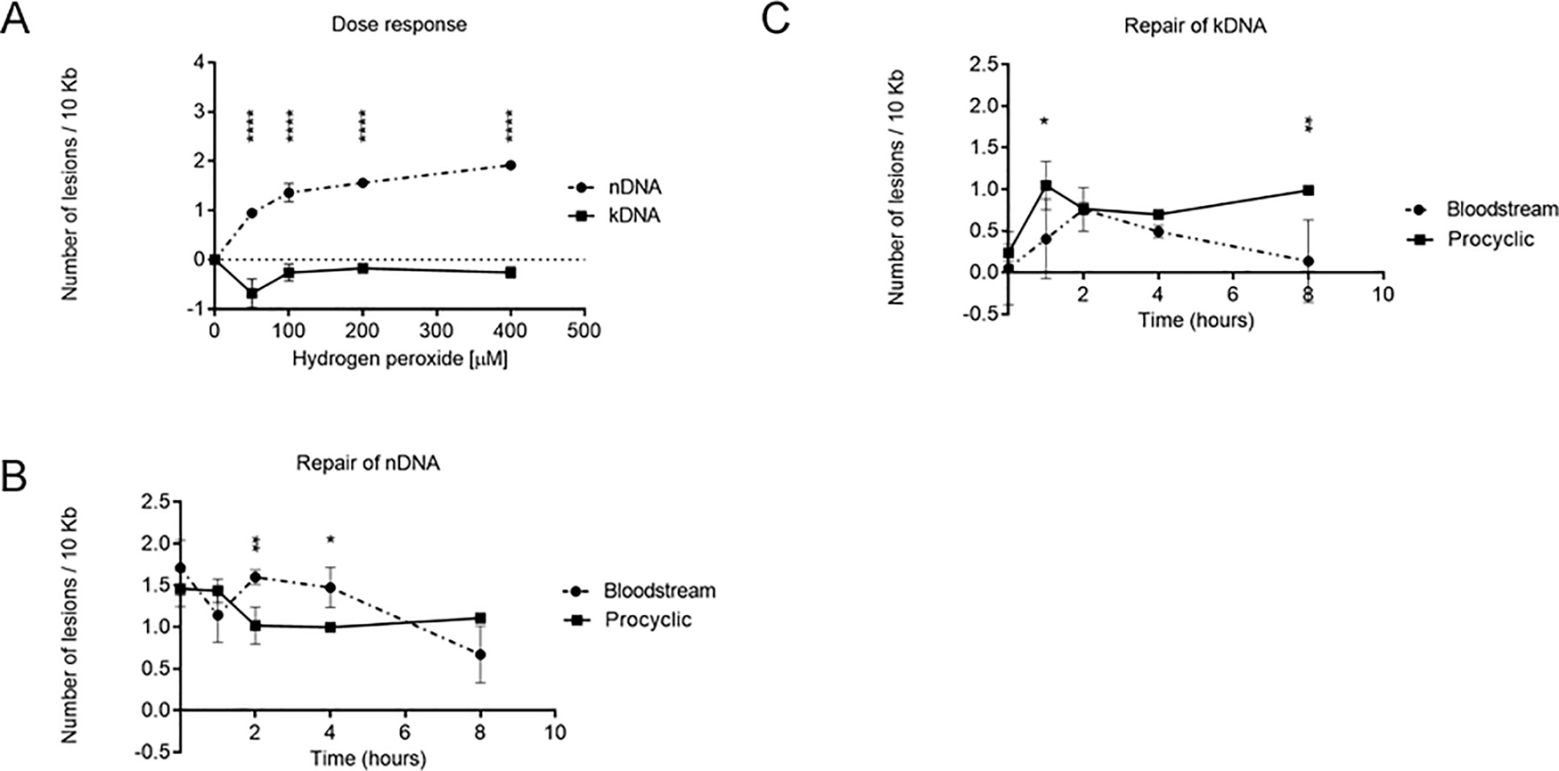
**Measurement of DNA damage and repair of lesions caused by hydrogen peroxide treatment. A)** Dose response curve of DNA lesions induced by hydrogen peroxide in nDNA (traced line with circles) and kDNA (full line with squares) of BSF cells. Cells were treated with 50, 100, 200, and 400 μM of hydrogen peroxide for 1 h. Repair kinetics of oxidative damage in nDNA **(B)** and kDNA **(C)** of BSF (traced lines with circles) and PCF (full lines with squares) cells treated with 100 μM of hydrogen peroxide for 1 h. Negative values of lesions indicate lower frequency of DNA damage relative to the untreated control. The data were obtained from two independent PCR amplifications derived from each of the two biological duplicates. Error bars denote standard deviation and *, **, and **** mean respectively p values less than 0.05, 0.01, and 0.0001 calculated by Two-way ANOVA repeated measures with fixed effects for cell type, time, and their interaction, followed by Sidak’s multiple comparisons post test.

Base excision repair (BER) tackles oxidative DNA damage in *T. brucei* [32], though further work suggests MMR may also act [[42], [43], [44]]. To induce oxidative damage and examine repair efficiency of the resulting lesions we exposed both life cycle forms of the parasite to hydrogen peroxide. To select an ideal dose we treated BSF cells with hydrogen peroxide concentrations ranging from 50 to 400 μM for 15 (data not show) and 60 min (Fig. 3A). No damage induction could be detected by PCR at any of the doses tested over 15 min (data not show). However, treatment with 100 μM hydrogen peroxide for one hour induced ∼1.5 lesions/10 kbp in the nDNA of both BSF and PCF cells (Fig. 3A, B), without significantly decreasing parasites survival 10 h after treatment (Fig. S1B). In contrast to cisplatin-induced damage, both BSF or PCF cells displayed relatively slow repair of oxidative damage in the nDNA: in each case reduction in the levels of PCR-blocking lesions occurred, but did not approach returning to basal levels of damage (Fig. 3B). Unlike in the nDNA, we could not detect damage induction in the kDNA of either BSF or PCF cells following exposure to hydrogen peroxide (Fig. 3A). However, increased levels of damage emerged in the time points following exposure (Fig. 3C), indicating that DNA damage induced by hydrogen peroxide in kDNA is caused indirectly. Moreover, such indirect damage induction arose more rapidly in PCF cells than in BSF, and was more slowly removed, since only in BSF cells did the levels of PCR-blocking lesions reduce in the kDNA to near basal levels during the time course analyzed (Fig. 3C).

### 3.3 BSF and PCF *T. brucei* cells display distinct patterns of induction and repair of lesions caused by MMS

**Fig. 4:**
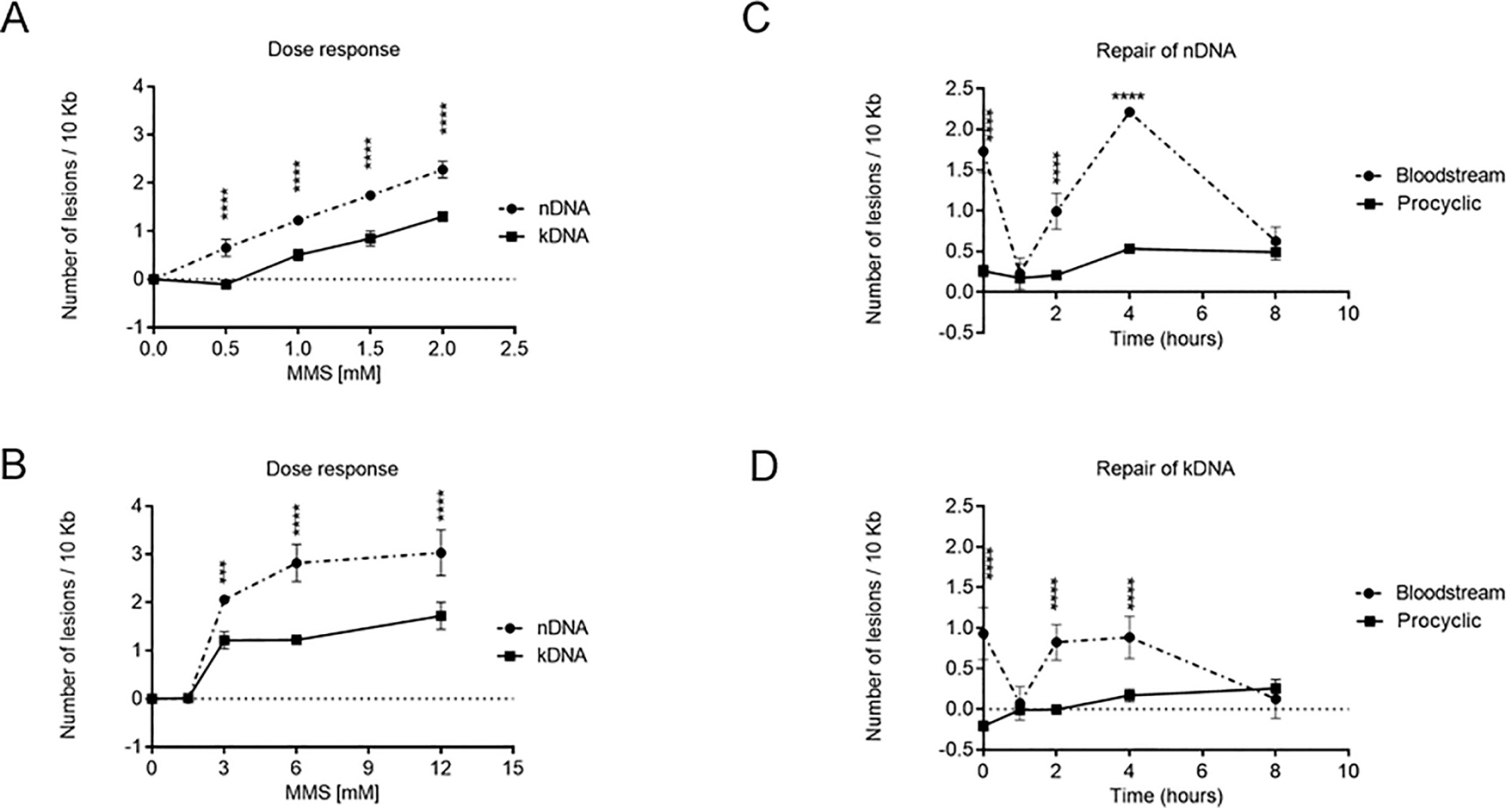
**Assessment of formation and repair of alkylating DNA damage in BSF and PCF cells treated with MMS.** Lesions frequencies in nDNA (traced lines with circles) and kDNA (full lines with squares) of BSF **(A)** and PCF **(B)** cells as a function of MMS dose (mM). BSF cells were treated with 0.5 to 2 mM of MMS for 1 h whereas PCF cells were treated with 1.5–12 mM of MMS for 1 h. DNA repair kinetics of MMS-induced lesions in nDNA (**C)** and kDNA **(D)** of BSF (traced lines with circles) and PCF (full lines with squares) cells treated with 1.5 mM of MMS for 1 h. The data were obtained from two independent PCR amplifications derived from each of two biological duplicates. Error bars denote standard deviation. *** and **** mean respectively p values less than 0.001 and 0.0001 calculated by Two-way ANOVA repeated measures with fixed effects for cell type, time, and their interaction, followed by Sidak’s multiple comparisons post test.

Since trypanosomatid NER targets cisplatin-induced adducts more efficiently than photolesions [29], and *T. brucei* repairs DNA oxidative damage with relatively low efficiency (Section 3.2), we next investigated how the parasite handles DNA alkylating damage. In most model organisms MMS damage is repaired by BER, though further repair pathways have also been implicated, including MMR and HR [55,56]. BSF and PCF cells displayed highly divergent sensitivity in the first hours after treatment with 1.5–6 mM MMS (Fig. S1C) and so, in order to select an appropriate dose to compare repair kinetics, we performed separate dose response curves in the two life cycle forms (Fig. 4A, B). Consistent with the different MMS sensitivities (Fig. S1C), we found that the pattern of MMS dose response, for both nDNA and kDNA, differed in BSF and PCF cells, and that different doses of MMS were necessary to induce similar levels of PCR-blocking lesions (Fig. 4A, B). A dose of 1.5 mM MMS for one hour induced ∼1.5 lesions/10 kbp in the nDNA of BSF (Fig. 4A), whereas 3 mM for one hour was needed to get the same lesion frequency in the nDNA of PCF cells (Fig. 4B).

The dynamics of MMS-induced lesion repair was markedly different in the two life cycle stages of *T. brucei* (Fig. 4A, B). BSF cells presented a complex pattern of repair, where the amount of PCR-blocking DNA damage in both nDNA and kDNA dropped rapidly within one hour after treatment, and then increased from 2 to 5 h, reaching a similar level of damage to that observed at the beginning of the recovery period. from 4 to 8 hours, the level of DNA damage then reduced again, returning to near basal levels 8 h after treatment. Thus, there appears to be two distinct phases of MMS-induced damage repair in BSF *T. brucei*: early repair, which occurs within the first hour after treatment, and late repair, which happens from 4 to 8 h (Fig. 4C, D). In striking contrast to the BSF cells, and consistent with the results obtained in the dose response curve, the treatment of PCF cells with 1.5 mM MMS does not induce detectable DNA damage either in nDNA or kDNA. However, there was a subtle increase in the level of lesions from 0 to 8 hrs (Fig. 4C and D). Taken together, these MMS data add to that described in Sections 3.1 and 3.2 and confirm substantial differences in DNA damage repair between BSF and PCF *T. brucei* cells, as well as strikingly different patterns of repair for the three forms of damage examined.

### 3.4 TbCSB is involved in the parasite response to MMS, while TbRad51 is required for the late processing of DNA alkylating damage

**Fig. 5:**
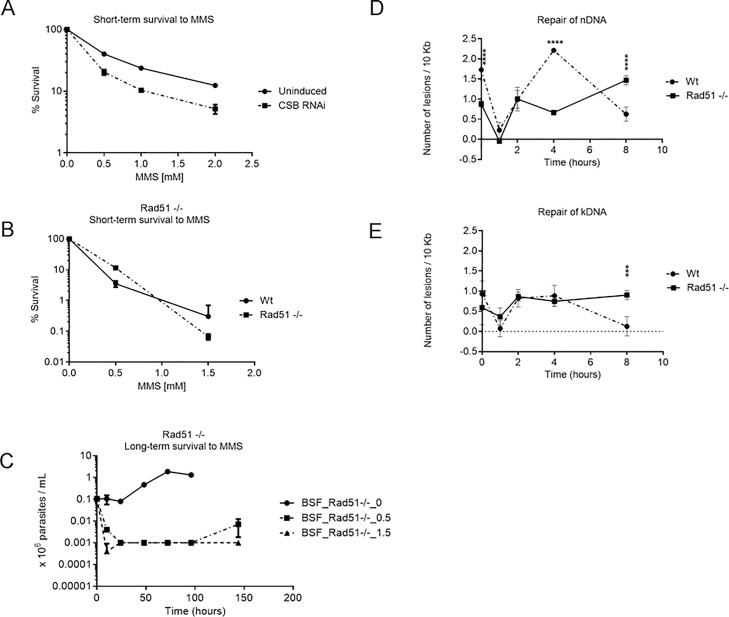
**CSB and RAD51 act in the response to *T. brucei* MMS-induced DNA damage. A)** Survival curve analysis of BSF cells 10 h after the treatment with 0.5–2 mM of MMS for 1 h in the presence and absence of TbCSB RNAi-mediated knockdown. **B)** Survival curve analysis of BSF *rad51* -/- cells 10 h after the treatment with 0.5–1.5 mM of MMS for 1 h. **C)** Analysis of BSF *rad51* -/- cells grown for 10 to 144 h after treatment with 0.5 and1.5 mM of MMS for 1 h. Values are the means of three independent experiments, and error bars denote standard deviation. DNA repair kinetics of MMS-induced lesions from nDNA **(D)** and kDNA **(E)** of BSF *rad51* -/- cells (full lines with squares) treated with 0.5 mM of MMS for 1 h. The data were compared and plotted together with results previously presented in Fig. 4 C, D of repair curves in nDNA and kDNA from BSF wild type (wt) cells (traced lines with circles) treated with 1.5 mM of MMS for 1 h. The data were obtained from two independent PCR amplifications derived from each of two biological duplicates. Error bars denote standard deviation. A Two-way ANOVA repeated measures with fixed effects for cell type, time, and their interaction followed by Sidak’s multiple comparisons post test were used to analyze the DNA repair data, p<0.01, 0.001 and 0.0001 (**, *** and ****).

One explanation for the complex pattern of DNA repair during the removal of MMS-induced damage in BSF cells is that two (or more) DNA repair pathways can remove DNA alkylating damage in *T. brucei*. Most cells studied to date repair MMS-alkylated bases by BER. However, the deletion of APE1, which plays a key role in BER by cleaving the sugar phosphate backbone at the 5’ end of an abasic site, does not increase BSF *T. brucei* sensitivity to MMS [32], suggesting a crosstalk between BER and other repair pathways. To ask whether other proteins than those enrolled in canonical BER could have a role in the repair of alkylating DNA damage in BSF *T. brucei*, we compared the sensitivity of BSF *T. brucei* cells to MMS before and after RNAi-mediated knockdown of TbCSB, a component of transcription-coupled NER [29]. Cells in which RNAi against CSB had been induced displayed increased sensitivity to MMS compared with uninduced cells at three concentrations of MMS when growth was evaluated in BSF cells in the 10 h following exposure (Fig. 5A), suggesting involvement of TbCSB in the repair of MMS-alkylation lesions in *T. brucei* BSF cells.

Unrepaired lesions or repair intermediates that persist until S phase might block DNA replication, leading to replication fork collapse and, potentially, DSB induction [56]. To test whether *T. brucei* HR contributes to the complex pattern of MMS repair, we evaluated the repair efficiency and sensitivity of a BSF RAD51 null mutant strain (*rad51* -/-) after induction of MMS damage. RAD51 absence altered the profile of nDNA lesion removal, appearing not to impede the early (up to 1 h) repair but to prevent the removal of lesions that accumulated later, suggesting HR acts in the late repair (Fig. 5D). Consistent with this, survival of *rad51*-/- cells up to 10 h after exposure to two concentrations of MMS was not notably different from wild type cells (Fig. 5B), but survival of the null mutants was drastically curtailed when growth was measured at the same MMS doses over 150 h (Fig. 5C). In the PCR repair assay (Fig. 5D), it was notable that nDNA lesions remerged from 1 to 2 hours in both the wild type and *rad51*-/- cells, suggesting either that some MMS lesions are not repaired during the first hour and accumulate thereafter, or the incomplete repair of these lesions will lead to more severe consequences, such as DSBs formation. Irrespective, the data are consistent with a role for RAD51 only in the late step of repair of nDNA lesions induced by MMS, perhaps when the BSF cells enter S-phase.

The above data reveal a role for RAD51 in the late repair of DNA alkylating damage in BSF nDNA. To evaluate whether RAD51 is also involved in repair in the kDNA of BSF cells, we monitored damage induction and repair by the kDNA PCR assay, treating *rad51* -/- cellswith 0.5 mM MMS for one hour. Here, the absence of RAD51 affected only the late step of the repair of MMS-induced damage, presenting a similar pattern to what we observed in the repair of nDNA (Fig. 5E). These data indicate a role for RAD51 in the maintenance of kDNA integrity in BSF *T. brucei*.

### 3.5 BSF *T. brucei* cells show higher sensitivity to DNA damage than PCF cells

**Fig. 6:**
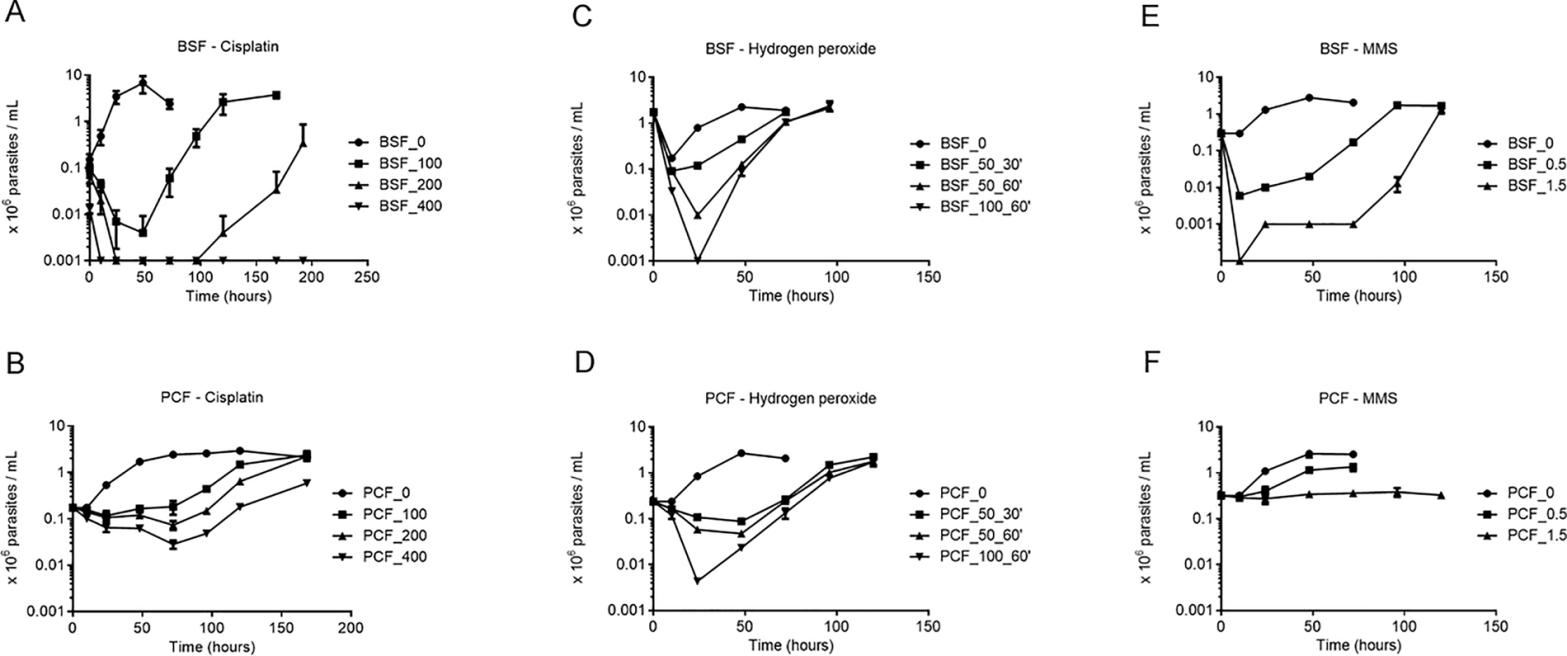
**Growth curves of BSF and PCF cells after treatment with cisplatin, hydrogen peroxide, and MMS. A)** BSF cells treated with 0 (circles), 100 (squares), 200 (triangles), and 400 μM (inverted triangles) of cisplatin for 1 h. **B)** PCF cells treated as described in (A). **C)** BSF cells treated with 0 (circles), 50 μM of hydrogen peroxide for 30 min (squares), 50 μM for 60 min (triangles), and 100 μM for 60 min (inverted triangles). **D)** PCF cells treated as described in (C). **E)** BSF cells treated with 0 (circles), 0.5 (squares), and 1.5 (triangles) mM of MMS for 1 h. **F)** PCF cells treated as described in (E). Data are the means of three independent experiments and error bars represent standard deviation.

In the data above (Sections 3.1 and 3.2) we show that exposure to cisplatin or hydrogen peroxide induces similar levels of DNA damage in both *T. brucei* life stages. However, BSF cells show higher efficiency than PCF cells in repairing cisplatin and hydrogen peroxide-induced DNA damage. Though higher doses of MMS were needed in PCF cells to induce similar levels of damage to that seen in BSF cells, there was greater evidence of active repair in the latter life cycle stage (Section 3.3). To test if the higher efficiency of BSF DNA repair is reflected in greater cell survival relative to PCF cells, we induced DNA damage using the time-limited exposure approaches employed in the DNA repair assays and then we evaluated recovery of cell growth. Despite the higher DNA repair efficiency of damage induced by the treatments with cisplatin and hydrogen peroxide, BSF cells displayed substantially higher cell death rates than PCF cells (Fig. 6A–D). The MMS treatment used induces higher level of DNA damage in BSF cells than in PCF, and resulted in hugely accentuated death and growth impairment in the former life cycle stage (Fig. 6 E, F). Thus, we conclude that BSF cells invariably display higher cell death rates regardless of the damage level or lesion type induced in the *T. brucei* genome(s).

### 3.6 BSF and PCF *T. brucei* cells undergo cell cycle arrest or perturbation after DNA damage

**Fig. 7:**
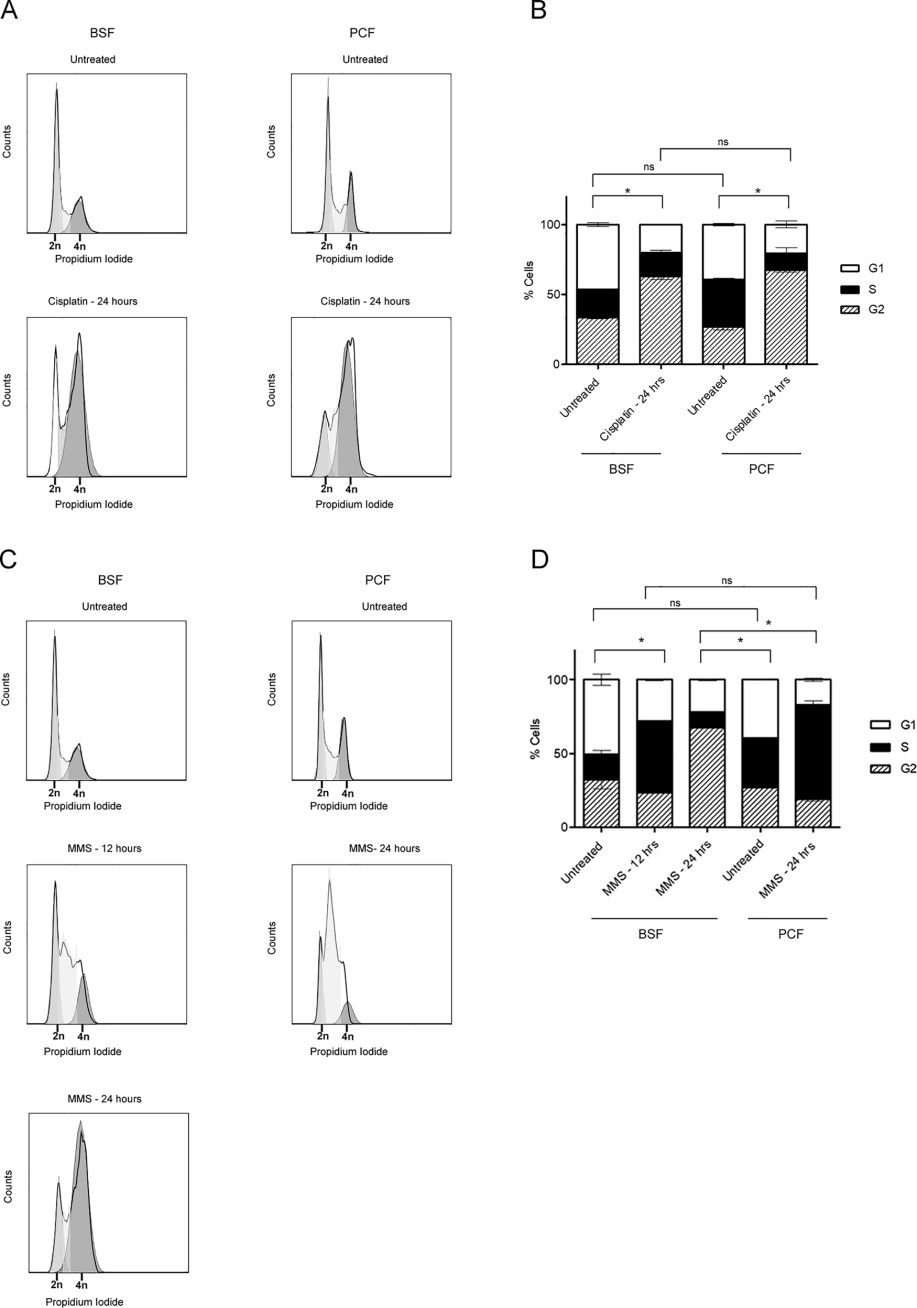
**Cell cycle analysis by flow cytometry of asynchronous log-phase BSF and PCF cell populations after DNA damage induction. A)** Representative histograms of BSF (left column) and PCF (right column) cells with no treatment (Untreated) and 24 h after treatment with 100 μM of cisplatin for 1 h (Cisplatin – 24 h). **B)** Quantification of (A). **C-)** BSF (left column) and PCF (right column) cells with no treatment (Untreated) and 12 and/or 24 h after treatment with 1.5 mM of MMS for 1 h (MMS – 12/24 h). **D-)** Quantification of (C). FACS analysis was performed on 10,000 cell counts (y axis). The channel FL2-A (x axis) was used to detect propidium iodide staining and therefore to quantify DNA content. Asterisks denote p values less than 0.05 calculated by chi-square test; n.s.= not significant.

BSF and PCF *T. brucei* cells present different DNA repair efficiencies and different cell survival rates upon damage induction by the three genotoxic agents examined. To test whether differential regulation of a DNA damage checkpoint would account for the distinct outcomes presented by BSF and PCF cells, we evaluated their cell cycle patterns by flow cytometry after exposure to MMS and cisplatin (Fig. 7). The cell cycle distribution of asynchronous populations of both life cycle stages were altered by both forms of DNA damage. After cisplatin treatment, both BSF and PCF cells displayed a loss of 2n cells and an increase in 4n, indicating both had accumulated in the G2/M phase of cell cycle (Fig. 7 A, B).

BSF and PCF cells both accumulated in S phase (DNA content between 2n and 4n) after MMS treatment. However, though BSF cells remained arrested in S phase for only 12 h, PCF cells displayed a longer S-phase accumulation, with increased 2n-4n DNA seen at 24 h. These results suggest that the timing of the cell cycle response to alkylation damage differs in the two life stages (Fig. 7C and D). In addition, though we are able to observe recovery of a normal cell cycle distribution of PCF cells after treatment with cisplatin and MMS (Fig. S3), we could not track BSF cell recovery by flow cytometry experiments because of their high death rates, further indicating a differing cell cycle response in the two life cycle forms.

Taken together, these data suggest that, despite differing repair and growth profiles after cisplatin DNA damage, BSF and PCF cells may activate similar putative checkpoint responses, though with differing long term consequences. In contrast, MMS damage elicits distinct repair, growth and cell cycle responses in the two life cycle stages. Thus, these data reveal differing DNA damage responses and, potentially, divergent cell fates in BSF and PCF cells after genome damage.

### 3.7 PCF and BSF *T. brucei* cells undergo distinct cell death pathways upon DNA damage induction

**Fig. 8:**
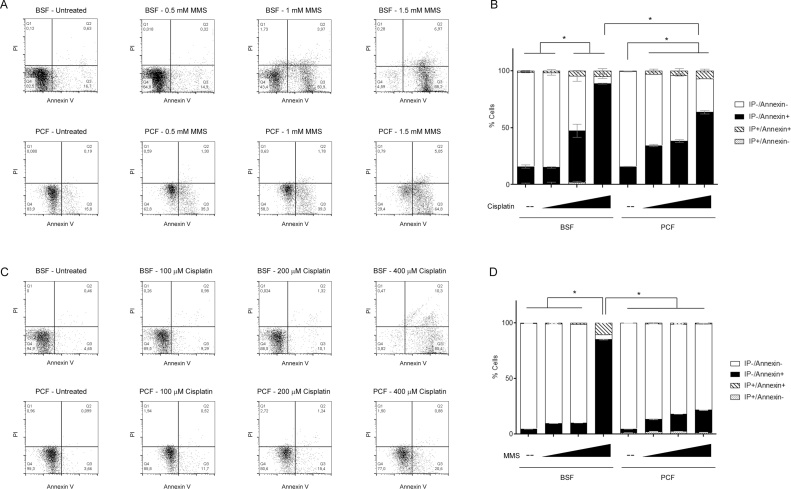
**Flow cytometry analysis of asynchronous log-phase populations in the presence and absence of DNA damage by FITC Annexin V and propidium iodide staining. A) Top row:** BSF cells with no treatment and immediately after exposure to 0.5, 1.0 or 1.5 mM of MMS for 1 h. **Bottom row:** PCF cells 24 h after treatment with 0. 1.0 or 1.5 mM of MMS for 1 h. **B)** Quantification of A-). **C)Top row:** BSF cells with no treatment and 24 h after treatment with 100, 200 or -400 μM of cisplatin for 1 h. **Bottom row:** PCF cells treated and analyzed as described in the top row. **D)** Quantification of C-). FACS analysis was performed on 5000 cell counts. Channels FL2-H (y axis) and FL1-H (x axis) were used to detect, respectively, propidium iodide (PI) and FITC-Annexin V (Annexin-V) staining. Asterisks denote p values less than 0.05 calculated by chi-square test.

Cell death in mammals can be classified as regulated or unregulated depending on several biochemical and morphological features. Regardless of the ongoing debate regarding whether or not trypanosomatids can induce cell death by apoptosis or programmed cell death, these organisms present more than one pathway of cell death, which have been classified as necrosis and incidental cell death, with the latter pathway sharing some features with intrinsic apoptosis, such as phosphatidylserine (PS) exposure, nuclear DNA fragmentation, and loss of mitochondrial membrane potential. Because the molecular machinery of intrinsic apoptosis has not been identified in trypanosomatids, this particular cell death mechanism is classified as incidental cell death and grouped together with all other cell death pathways which differ from unregulated necrosis [57]. To ask if different cell death pathways arise in BSF and PCF *T. brucei* cells after DNA damage induction we monitored parasite cell death through propidium iodide (PI) and FITC Annexin V staining after treatment with MMS and cisplatin. Annexin V has strong affinity to phosphatidylserine (PS), which is present in the outer face of the plasma membrane of cells undergoing apoptosis. Therefore, we classified FITC Annexin V positive cells as undergoing incidental cell death and double positive cells (Annexin V and PI-positive) as necrotic cells, whereas double-negative cells indicates vital cells [58]. After both types of damage, BSF cells presented greater PS exposure than PCF cells, suggesting higher occurrence of incidental cell death in the former than in the latter (Fig. 8). Thus, the data suggest greater levels of cell death associated with PS exposure in BSF cells than in PCF upon induction of DNA damage, which may be explained by the differing growth responses of the two cell types, as well as by the differing cell cycle changes after MMS exposure.

## 4 Discussion

In this study, we have analyzed *T. brucei* DNA repair in both genomes of the parasite (nDNA and kDNA), and in two replicative life stages after DNA damage induction. The data reveal active repair of genome damage in both organelles, though repair kinetics were not equivalent in the two genomes. In addition, strikingly different repair kinetics for DNA damage are seen in the two life cycle stages examined, as well as diverged growth responses, some of which can be explained by variant cell cycle responses. After three forms of damage - oxidation, alkylation and DNA crosslinking - mammal-infective *T. brucei* cells display greater cell death than tsetse-infective cells.

Cisplatin, hydrogen peroxide and MMS generate distinct forms of DNA damage, which appear to be tackled in different ways in *T. brucei*. The parasite appears to be highly efficient in repairing cisplatin-induced DNA damage, as the kinetics of lesion removal were markedly fastest for this genotoxic agent. Indeed, cisplatin damage repair in *T. brucei* appears faster than in human cells, which (when analyzed using the same PCR technique) do not remove more than 50% of cisplatin adducts present in a transcriptionally active gene within 24 h [59]. One explanation is that failure to rapidly tackle DNA cross-links would be highly deleterious to *T. brucei*, given its near universal use of multigenic transcription. In contrast to cisplatin, though *T. brucei* can repair oxidative damage, the efficiency of the reaction is lower, which again contrasts with human fibroblasts, which can repair most oxidative damage in nDNA within 1.5 h [60]. Finally, we found that BSF *T. brucei* presents a complex pattern of repair of MMS-induced damage. This pattern may correspond with the rapid DNA synthesis inferred by microfluorometry analysis of nDNA and kDNA in *T. b. gambiense* treated with bleomycin [61]. Though bleomycin can cause DSBs, it can also induce abasic sites, the first intermediate that occurs after removal of an alkylated base [62]. In contrast to BSF cells, PCF cells were resistant to higher levels of MMS, which might suggest PCF cells possess a more effective detoxification mechanism or perhaps can directly revert alkylating DNA damage.

The number of lesions induced after exposure to MMS, cisplatin or hydrogen peroxide in kDNA, within the same parasite life stage, was always lower than in nDNA. Moreover, the repair of cisplatin-induced adducts in kDNA appeared more effective than in nDNA. In this regard, *T. brucei* presents the opposite pattern of nuclear versus mitochondrial DNA repair when compared to human cells, where mitochondrial DNA lesions caused by hydrogen peroxide are more extensive and persistent than nuclear lesions localized [60]. Though lesions caused by MMS are not more extensive in human mitochondrial DNA relative to nuclear DNA, they last much longer [63], whereas MMS-induced lesions appear to be tackled at similar rate in the nucleus and kinetoplastid of *T. brucei*. Thus, *T. brucei* appears to encode efficient kDNA repair. Though mitophagy or DNA ejection resulting from kDNA damage has not been described in *T. brucei*, viable akinetoplastic/diskinetoplastic (total/partial loss of kDNA) BSF cells can be induced by the treatment with DNA intercalators [64]. However, akinetoplastic/diskinetoplastic BSF cells are only viable due to compensatory mutations in the nuclear encoded F1/F0 ATPase subunit γ [65]. In insect-derived forms, kDNA loss induced by chemical treatment results in non-viable parasites [64]. Therefore, the maintenance and repair of kDNA seems to play an essential role during most, if not all, of the parasite life cycle. Indeed, the *T. brucei* nDNA and kDNA repair curves were very similar for some cisplatin and MMS damage, suggesting multiple DNA repair pathways act to promote stability of both genomes. Trypanosomatid kDNA is an interlinked network of minicircle and maxicircle concatemers, which requires several proteins for its maintenance, most of which have been associated with replication [48]. Information regarding what DNA repair pathways are present in trypanosomatid mitochondria is more limited and, based on the work here, merits further investigation. For instance, no study has so far attributed a role for RAD51 in trypanosomatid kDNA metabolism, in contrast to several studies describing the role of RAD51 in parasite nDNA repair [13,14,51,66]. Though immunofluorescence has only detected RAD51 in the nucleus of *T. brucei* [14,51,66], the PCR assay here demonstrates impaired repair of MMS damage in the kDNA of RAD51 mutants. What role TbRAD51 provides is unclear, but recombination-assisted DNA replication is a common theme of catenated DNA [67], such as mitochondrial DNA from yeast [68] and human cardiac cells [69].

NER repairs helix distorting DNA lesions and is subdivided in GG-NER, which targets DNA lesions genome wide, and TC-NER, which targets DNA damage that impedes transcription [70]. As noted above, transcription in trypanosomatids is unusual, in that virtually all genes are encoded initially as multigene transcripts, meaning most of the genome is traversed by RNA polymerase. Consistent with this, *T. brucei* NER shows specialization because, amongst the genes tested for function, only the TC-NER genes TbCSB, TbXPBz and TbXPG have been shown clearly to act in NER. In contrast, the GG-NER genes TbXPC and TbDDB appear to function in inter-strand cross link repair [29]. This NER specialization probably explains the rapidity by which cisplatin adducts are repaired, with TbCSB likely to act as the main regulator, evoking TC-NER-mediated repair of cisplatin lesions that block RNA Polymerase [29]. In fact, the involvement we now describe for TbCSB in tackling MMS damage suggests that maintaining transcription is crucial, with TbCSB having been co-opted to repair alkylation DNA damage. Alkylated bases are classically targeted by BER through the action of DNA glycosylases, thus generating abasic sites that, if handled by short-patch BER, will be processed by the sequential action of an AP endonuclease (APE1), DNA polymerase β, and a DNA ligase [55]. However, mutation of APE1 in BSF *T. brucei* does not change the parasite’s sensitivity to MMS and temozolomide [32], suggesting BER adaptations in trypanosomatid repair of alkylated bases. In this case, one possible hypothesis is that TbCSB could promote the repair of transcription-blocking BER intermediates by recruiting the BER scaffold protein XRCC1, as suggested to occur in human cells [71], thus facilitating the repair of damaged bases. However, no XRCC1 homolog is found in trypanosomatids, perhaps suggesting other mechanistic adaptations. Alternatively, alkylating DNA damage can be repaired by NER itself, since 8-oxoguanine can be removed by TC-NER [72]. Moreover, CSB can stimulate several DNA glycosylases [73] and APE1 [74], and yeast NER can tackle abasic sites [75]. Thus, there is precedence for crosstalk between BER and NER and it is possible this has assumed greater prominence in trypansomatids due to their novel transcription strategies.

Excision repair intermediates, such as abasic sites or gaps, are even more toxic than the initial lesion, because they cause collapse of the replication fork, perhaps leading to the formation of DSBs [56]. The absence of RAD51 in BSF *T. brucei* impacts only on the late steps of MMS-induced nDNA damage repair, with lesions accumulating from 4 to 8 hours rather than being removed (as occurs in wild type cells). This effect, allied to the pronounced increase of MMS- induced lesions in wild type BSF cells from 2 to 5 hours relative to *rad51* -/- cells suggests a complex picture of alkylation repair in these cells. A feasible model is that in wild type cells alkylating DNA damage is tolerated if incompletely repaired until DNA replication occurs, when RAD51 promotes tolerance in S phase. After replication, most of the damage is successfully repaired either by excision repair or HR. In RAD51 knockout cells, HR capacity is presumably severely impaired. Therefore, excision repair which is otherwise counteracted by RAD51, takes place at S phase and causes a delay in DNA replication. After S phase, and in the absence of HR, under-replicated sites arising from impaired DNA replication will contribute to DSBs formation and genomic instability. Consistent with this model, RAD51 from yeast prevents the repair of gaps left behind the replication fork to avoid spurious HR reactions in S phase, but assures proper HR-based repair of these gaps after DNA replication and S phase are concluded [76]. Therefore, such a mechanism of damage tolerance performed by HR in yeast and other eukaryotic lineages [77] might be also conserved among trypanosomatids.

In addition to the differing strategies of DNA protection and repair used by BSF and PCF cells in response to damage induced by alkylation, DNA repair of cisplatin and hydrogen peroxide lesions, which are potentially repaired by NER and BER, is more efficient in BSF *T. brucei* than in PCF. Regulation of DNA repair is well established when the choice of repair pathway used depends on the cell cycle phase. Upon DSB formation, for example, NHEJ is predominantly enacted when a yeast or mammalian cell is in the G1 phase, while HR predominates in S/G2 phases [78]. In contrast, DNA repair regulation depending on cell type or cell differentiation is less understood. However, mammalian embryonic stem cells are known to repair DSBs through HR and present a high capacity to perform NER and BER pathways, whereas post-mitotic cells use NHEJ to repair DSBs and downregulate excision repair [79,80]. PCF and BSF T. brucei cells present different cell cycle checkpoints even in non-damaging conditions: PCF cells can proceed to cytokinesis despite a blockage in S phase or mitosis, but in BSF cells inhibition of mitosis impedes cytokinesis but cannot block new rounds of DNA replication [9,10]. How these checkpoints relate to DNA damage conditions has been little examined, despite recent descriptions of multiple protein kinases that respond to MMS-induced damage [49]. Both life cycle forms of *T. brucei* present similar patterns of G2/M cell cycle arrest following cisplatin exposure, perhaps indicating they share the same mechanism of checkpoint signaling. In contrast, though both BSF and PCF cells accumulate in S-phase after MMS treatment, BSF cells remain arrested in this cell cycle phase for less time. In response to both forms of damage, cell death appears to be more pronounced in BSF cells (as determined by growth and flow cytometry), whereas PCF cells appear to show a greater capacity for cell growth arrest. Protein networks involved in sensing and signaling DNA damage, thus halting the cell cycle and dictating cell fate, can vary amongst distinct cell types belonging the same organism. Mammalian embryonic stem cells, for instance, present an unusual bypass of the G1 checkpoint and a lower threshold to trigger apoptosis, while post-mitotic cells have a fully active checkpoint and restrict apoptosis [79,80]. *Caenorhabditis elegans* also pursues differential patterns of response to DNA damage, with germ line cells having higher efficiency DNA repair and being more prone to undergo apoptosis than somatic cells [81,82]. Taken together, we hypothesize that BSF *T. brucei* may share more features of the DNA damage response with stem cells, while PCF *T. brucei* more resemble the strategies of adult post-mitotic cells to cope with DNA damage. We suggest that BSF cells are programmed to perform fast DNA replication by deactivating at least some checkpoint signaling. To compensate for this checkpoint deactivation, and to avoid the deleterious effects of replicative stress, BSF cells upregulate DNA repair. However, in conditions of extreme DNA damage repair can be overwhelmed, with cells accumulating in G2/M as they encounter unrepaired blocks to replication, which are then lethal as the cell continues to undergo mitosis. It is conceivable that this strategy stems from growth requirements in the mammal: if VSG switching is intimately associated with DNA replication, then there will be pressure to prioritize replication in order to sustain the infection in the face of the mammalian immune response. In contrast, upon differentiation to the PCF, *T. brucei* appears to reactivate DNA damage checkpoint(s), allowing replication to be paused until DNA repair is accomplished. Here, cell death or senescence would be less frequent and only occur when DNA repair fails to restore genome integrity.

In summary, we suggest that developmental regulation of the DNA damage response in *T. brucei* can account for the different repair phenotypes described here for BSF and PCF cells, and for the differing repair and growth phenotypes of DNA repair gene mutants in the two life cycle stages [43,50,51]. To date, differential use of the DNA damage response has mainly been considered in the context of cell differentiation during the development of multicellular organisms [79,80,82]. We show here that the DNA damage response can be varied depending on the developmental stage of a unicellular organism. We cannot trace a direct parallel between the molecular mechanisms of DNA damage response regulation from *T. brucei* to multicellular organisms, despite extensive conservation of the *T. brucei* DNA repair machinery, because of lack of conservation of canonical mechanisms of programmed cell death in the protozoan parasite [57], as well as limited understanding of the signaling events that co-ordinate DNA repair during the *T. brucei* cell and life cycle. Further studies will be necessary to determine the cellular mechanisms that regulate the DNA damage response in *T. brucei* and related, early branching eukaryotes.

## 5 Conclusions

Mammal and insect-derived *T. brucei* life cycle cells present different DNA repair efficiencies and distinct cellular responses to DNA damage, indicating developmental regulation of the DNA damage response. DNA repair is active in the *T. brucei* mitochondrial genome, with similar kinetics to nuclear repair. RAD51 promotes maintenance of the *T. brucei* mitochondrial genome.

## Funding

Work in Glasgow was supported by the Wellcome Trust [089172] and the BBSRC [BB/K006495/1, BB/M028909/1, BB/N016165/1]. The Wellcome Centre for Molecular Parasitology is supported by core funding from the Wellcome Trust [104111]. Work in Brazil was supported by FAPEMIG and CNPq.

## Conflictofinterest

None declared.
