# Supplementary material for: The DNA damage response is developmentally regulated in the African trypanosome
Source: DNA Repair (Amst). 2019 Jan;73:78–90. doi: 10.1016/j.dnarep.2018.11.005 (PMC6329875; doi:10.1016/j.dnarep.2018.11.005)
Supplement: Supplementary file 1 [file mmc1.docx]

**Figure S1: Survival fractions of BSF (full lines with circles) and PCF (traced lines with squares) cells relative to untreated control 10 hours (short-term survival) after DNA damage induction. A)** Treatment with 100, 200, and 400 μM of cisplatin for 1 hour. **B)** Treatment with 100, 200, and 400 μM of hydrogen peroxide for 1 hour. **C)** Treatment with 1.5, 3, and 6 mM of MMS for 1 hour. Data points represent means of three independent experiments performed in triplicates. Error bars denote standard deviation.

**Figure S2: Induction and repair of DNA damage induced by UVC light. A)** Dose response curve of DNA lesions induced by UVC in nDNA (traced line with circles) and kDNA (full line with squares) of BSF cells. Cells were treated with 50, 100, 250, and 500 J/m^2^ of UVC light (254 nm). Repair kinetics of photolesions in nDNA **(B)** and kDNA **(C)** of BSF (traced lines with circles) and PCF (full lines with squares) cells treated with 30 J/m^2^ of UVC. The data were obtained from two independent PCR amplifications derived from each of the two biological duplicates. Results were analyzed using Two-way ANOVA repeated measures with fixed effects for cell type, time, and their interaction. This analysis was followed by Sidak’s multiple comparisons post test. Error bars denote standard deviation and ***, and **** mean respectively p values less than 0.001, and 0.0001 for comparisons between BSF versus PCF time points and ****^a^ means p<0.0001 for comparisons made between the point “0” after damage induction versus each subsequent time point of DNA repair. **D)** Survival analysis of BSF (full lines with circles) and PCF (traced lines with squares) cells relative to untreated control 10 hours (short-term survival) after irradiation with 30, 60, and 120 J/m^2^ of UVC light. Data points represent means of three independent experiments performed in triplicates. Error bars denote standard deviation.

**Figure S3: Cell cycle analysis by flow cytometry of asynchronous log-phase PCF cell populations recovering from DNA damage induction. A)** Cells with no treatment (Untreated) and 24, 48, and 72 hours after treatment with 100 μM of cisplatin for 1 hour (Cisplatin – 24, 48, and 72 hours). **B)** Cells with no treatment (Untreated) and 24, 48, 72, and 96 hours after treatment with 1.5 mM of MMS for 1 hour (MMS – 24, 48, 72, and 96 hours). FACS analysis was performed as described in Figure 7. **C**) Quantification of (A). **D)** Quantification of (B). Asterisks denote p values less than 0.05 calculated by chi-square test. **E)** Counts of PCF cell populations analyzed in (A) and (B). Data represent three independent experiments performed in triplicates. Error bars denote standard deviation.
